# Social and environmental enrichment has different effects on ethanol and sucrose consumption in mice

**DOI:** 10.1002/brb3.767

**Published:** 2017-07-22

**Authors:** Joan Y. Holgate, Hilary Garcia, Susmita Chatterjee, Selena E. Bartlett

**Affiliations:** ^1^ Institute of Health and Medical Innovation Translational Research Institute Queensland University of Technology Woolloongabba QLD Australia; ^2^ Ernest Gallo Clinical and Research Center University of California San Francisco CA USA

**Keywords:** consumption, environment enrichment, ethanol, social enrichment, sucrose

## Abstract

**Background:**

Factors leading to the harmful consumption of substances, like alcohol and sucrose, involve a complex interaction of genes and the environment. While we cannot control the genes we inherit, we can modify our environment. Understanding the role that social and environmental experiences play in alcohol and sucrose consumption is critical for developing preventative interventions and treatments for alcohol use disorders and obesity.

**Methods:**

We used the drinking in the dark two‐bottle choice (2BC) model of ethanol and sucrose consumption to compare male C57BL/6 mice housed in the IntelliCage (an automated device capable of simultaneously measuring behaviors of up to 16 mice living in an enriched social environment) with mice housed in standard isolated and social environments.

**Results:**

Consistent with previous publications on ethanol‐naïve and ‐experienced mice, social and environmental enrichment reduced ethanol preference. Isolated mice had the highest ethanol preference and IntelliCage mice the least, regardless of prior ethanol experience. In mice with no prior sucrose experience, the addition of social and environmental enrichment increased sucrose preference. However, moving isolated mice to enriched conditions did not affect sucrose preference in sucrose‐experienced mice.

**Conclusions:**

The impact of social and environmental enrichment on ethanol consumption differs from sucrose consumption suggesting that interventions and treatments developed for alcohol use disorders may not be suitable for sucrose consumption disorders.

## 1 INTRODUCTION

Harmful consumption of substances, like alcohol and sugar, remain a world‐wide health issue with few effective interventions and treatments available. In 2012, 3.3 million deaths were attributed to alcohol consumption and 3.4 million deaths to being overweight or obese in 2010 (WHO, 2014). It is well known that the development of disorders, like alcohol use disorders (AUDs) and obesity, involves a complex interaction of genetic and environmental factors (for reviews see, Clarke et al., 2008; Enoch, 2006; Alegria‐Torres, Baccarelli, & Bollati, 2011; Sinha & Jastreboff, 2013). While we have little control over the genes we inherit, we do have the ability to modify our environment and as such, understanding the role that social and environmental experiences play in the consumption of alcohol and sugar is critical for developing preventative interventions and treatments.

In AUDs, daily life experiences (like people, places, smells, meal rituals, and stress) form cues which become associated with alcohol consumption, and subsequent exposure to these cues increases the amount of alcohol consumed during drinking bouts and impedes the ability to remain abstinent (for reviews see, Breese, Sinha, & Heilig, 2011; Little et al., 2005; Sinha & Li, 2007). Similarly, environmental cues can also influence the consumption of sugary foods (Burger & Stice, 2014; Grenard et al., 2013; Hattersley, Irwin, King, & Allman‐Farinelli, 2009; Schmidt, Voorn, Binnekade, Schoffelmeer, & De Vries, 2005). To date, the majority of preclinical research investigating the impact of social and environmental enrichment on ethanol and sucrose consumption has been directed toward ethanol consumption (see Tables 1 and 2). While there is considerable methodological variation between the models used in these studies (species, strain, ethanol concentration, type and length of ethanol access), they generally show that social deprivation (individual rather than group housing) increases ethanol consumption (Lodge & Lawrence, 2003; Schenk, Gorman, & Amit, 1990; Wolffgramm, 1990; Wolffgramm & Heyne, 1991). The role of other types of environmental enrichment designed to provide physical and/or cognitive stimulation is less clear. Studies in two different rat strains suggest that providing a combination of social and environmental enrichment causes an increase in ethanol consumption (Rockman, Gibson, & Benarroch, 1989; Rockman & Glavin, 1986). In contrast, another study found that environmentally enriched‐socially deprived and socially enriched‐environmentally deprived female C57BL/6 mice consumed less ethanol than socially and environmentally deprived counterparts (Pang et al., 2013). Interestingly, the studies by Rockman and colleagues (Rockman et al., 1989) and Pang and colleagues (Pang et al., 2013) found no difference in ethanol consumption between socially enriched‐environmentally deprived and environmentally enriched‐socially deprived rats and mice, respectively. While there are differences in sex, strain, and methodology in the above studies, which could explain the contrasting effects of combined social and environmental enrichment on ethanol consumption, it is established that social and environmental factors are important determinants of ethanol consumption in rodents.

**Table 1:** Social and environmental interaction and ethanol consumption

Species	Strain	Sex	Ethanol %	Method	Results	Study
Mouse	C57BL/6	Both	5	1‐bottle, 45 min, 1 day	♂: I < S^a^	Logue, Chein, Gould, Holliday, & Steinberg (2014)
Mouse	C57BL/6	Both	15	2BC, 2 hr, 14 days	Early ♂: I > S, ♀: I > S^b^ Late ♂: I = S, ♀: I < S	Lopez, Doremus‐Fitzwater, & Becker (2011)
Mouse	C57BL/6	♀	10	2BC, 24 hr, 6 weeks	I > S = EI	Pang et al. 2013)
Mouse	C57BL/6	♂	15	2BC, 2 hr, 6 weeks	I < S	Sanna et al. (2011)
Rat	Sprague Dawley	♂	9	Intermittent 2BC, 6 hr, 35 days	E > I = I to E > EtoI	Rockman et al. (1989)
Rat	Wistar	♂	9	Intermittent 2BC, 24 hr, 36 days	E > I	Rockman et al. (1986)
Rat	Wistar	♂	8–10	1‐bottle, 24 hr, 10 days or 2BC, 12 hr, 6 days	1‐bottle: S > I 2BC: S = I	Juarez & Vazquez‐Cortes (2003)
Rat	Wistar	♂	8	2BC, 24 hr, 24 days	S = I	Thorsell, Slawecki, & Ehlers (2005)
Rat	Fawn Hooded	♂	5	2BC, 24 hr, 4 weeks	S(NP) < I + S(P)	Lodge & Lawrence (2003)
Rat	Wistar	♂	20	2BC, 24 hr, 9 months	S < C < I StoI>I	Wolffgramm (1990)
Rat	Long Evans	♂	10	Intermittent 2BC, 24 hr, 37 days	Early: S < I^b^ Late: S = I	Schenk et al. (1990)

S, group housing; I, individual housing; EI, individual housing and enrichment; E, group housing and enrichment; S(P), alcohol preferring in group housing; S(NP), nonalcohol preferring in group housing; C, sensory without physical contact in single housing.

a Effect significant for bout duration in juvenile but not adult mice.

b Housed individually from P21 (Early) or P60 (Late).

**Table 2:** Sucrose consumption and social and environmental enrichment

Species	Strain	Sex	Sucrose %	Method	Results	Study
Mouse	CD1 Swiss	Both	5–10	1‐bottle, 30 min, 1 day	S = I	Moles & Cooper (1995)
Rat	Lister Hooded	♂	0.7–34	2BC, 30 min, once/week, 2 weeks	EI < S	Hall et al. (1997)
Rat	Sprague Dawley	♂	32	2BC, 48 hr, 2 days	S = I	Brenes & Fornaguera (2008)
Rat	Long Evans	♂	10	Operant FR1, 10 days	S = I = EI = ES	Grimm et al. (2013)

S, group housing; I, individual housing; EI, individual housing with enrichment; ES, group housing with enrichment.

Fixed ratio 1 (FR1) means the rat had to press a lever one time to receive access to one volume of sucrose reward.

In recent years, it has been shown that the consumption of sucrose can lead to behavioral changes which meet addiction criteria defined in the Diagnostic and Statistical Manual V (DSM‐V) (American Psychiatric Association, 2013) (for reviews see, Hoebel, Avena, Bocarsly, & Rada, 2009; Avena, 2007; Avena, Rada, & Hoebel, 2008), and influences the same reward pathways in the brain as substances of abuse, like nicotine (De Vries, de Vries, Janssen, & Schoffelmeer, 2005), ethanol (Steensland et al., 2010), opioids (Spangler et al., 2004), and cocaine (Lenoir, Serre, Cantin, & Ahmed, 2007). While many of the characteristic of sucrose consumption are like ethanol consumption, very little research has been directed toward investigating the role of social and environmental factors in sucrose consumption (see Table 2). With the exception of one study (Grimm et al., 2008) focusing on relapse behavior rather than the development and maintenance of sucrose consumption; the currently available research suggests neither social nor environmental nor a combination of these factors influence sucrose consumption (Brenes & Fornaguera, 2008; Hall, Humby, Wilkinson, & Robbins, 1997; Moles & Cooper, 1995). It remains, however, undetermined whether moving rats from deprived environments to enriched environments and vice versa impacts subsequent sucrose consumption, similar to ethanol consumption. If indeed social and environmental factors are not important for specific aspects of sucrose consumption behavior, this feature would make sucrose consumption unique to other substances of abuse, like morphine, cocaine, nicotine, and ethanol (Wolffgramm, 1990; Alexander, Beyerstein, Hadaway, & Coambs, 1981; Gipson, Beckmann, El‐Maraghi, Marusich, & Bardo, 2011; Mesa‐Gresa, Ramos‐Campos, & Redolat, 2013), and could be utilized to, not only, improve our understanding and treatment of AUDs (and other addictions), but also to enhance our understanding of the factors which drive excessive sucrose consumption and our ability to prevent and treat obesity.

In this study, we used the drinking in the dark (DID) two‐bottle choice (2BC) procedure (Rhodes, Best, Belknap, Finn, & Crabbe, 2005) to model ethanol and sucrose consumption in mice and compared the effect of social (S) and social plus environmental enrichment (E) with social and environmental deprivation (I). The DID 2BC procedure is most commonly used to model excessive binge‐like ethanol consumption in mice for studying the underlying mechanisms of ethanol consumption disorders and identifying potential novel therapeutics (e.g., see Fritz et al., 2014; Gritz, Larson, & Radcliffe, 2016; Patkar et al., 2017). More recently, it has been adapted for studying excessive binge‐like sucrose consumption (e.g., see Steensland et al., 2010; Patkar et al., 2017; Dhaher et al., 2009). It should be noted that the model of sucrose consumption described in this study is different to the model of sucrose consumption commonly used for measuring depression‐like behaviors. The later model generally involves administering lower concentrations of sucrose to mice for short periods (once weekly or for a few days at a time) (e.g., see Benturquia et al., 2007; Covington, Vialou, LaPlant, Ohnishi, & Nestler, 2011; Ho et al., 2016). However, the DID model generally uses higher sucrose concentrations which are administered for long periods of time (daily for weeks to months) (e.g., see Fritz et al., 2014; Gritz et al., 2016; Patkar et al., 2017). Models utilizing long‐term administration of sucrose are not suitable for measuring depression‐like behaviors as they produce morphological changes within the brain (Klenowski, Shariff, et al., 2016). As the space available for free movement in the standard mouse cage was limited during standard group housing conditions, the addition of enrichment to the standard sized cage, which would have further reduced the living space available, was undesirable. Living in cramped conditions creates stress and could potentially confound any positive effects obtained from the provision of enrichment (Lin et al., 2015). To avoid this issue, we used the IntelliCage (NewBehaviour) to provide environmental enrichment in a social setting while measuring ethanol or sucrose consumption with the DID procedure. The IntelliCage is a novel automated instrument, capable of continuously tracking the movements of up to 16 mice simultaneously and has sufficient space to include enrichment objects without compromising living space availability. Finally, we explored whether the beneficial effects obtained from the provision of social and environmental enrichment could outweigh the biological effects of ethanol and sucrose by modifying the housing conditions of the mice and comparing subsequent ethanol and sucrose consumption.

## 2 MATERIAL AND METHODS

### 2.1 Drugs

Five percent (w/v) sucrose (Sigma, St. Louis, USA) and 20% (v/v) ethanol solutions (Gold Shield Chemical Ac., California, USA) were prepared in filtered water.

### 2.2 Animals and housing

Male 5‐week‐old C57BL/6 mice (Jackson Laboratories, California, USA or Animal Resource Center, Australia) were given at least 5 days to acclimatize to the new housing environment before experiments commenced. The mice were housed on a 12 hr reverse light cycle in a climate controlled room and had free access to food and water at all times. The experimental procedures followed the ARRIVE guidelines and were approved by the animal ethics committee at the Ernest Gallo Clinic and Research Centre, University of California, San Francisco (California, USA), University of Queensland (Brisbane, Australia), and Queensland University of Technology (Brisbane, Australia) in accordance with National Institutes of Health (NIH) and National Health and Medical Research Council (NHMRC) guidelines for the care and use of laboratory animals.

### 2.3 Drinking in the dark (DID) 2BC

**Figure 1:**
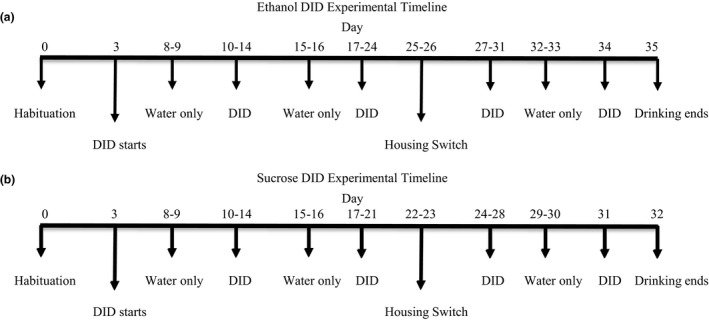
Experimental timeline. Following a habituation period, mice housed individually, in groups of 3–5 or in groups of 12 in the IntelliCage, consumed 20% (v/v) ethanol for 18 sessions (a) or 5% (w/v) sucrose for 15 sessions (b) using the drinking in the dark paradigm. Immediately following the last drinking session, the housing environments were changed. Approximately 3 days later, the mice recommenced drinking sessions for seven more sessions. After every fifth session, mice has access to water only for 2 days

**T**he DID model (Rhodes et al., 2005; Santos, Chatterjee, Henry, Holgate, & Bartlett, 2013) was originally adapted from [Rhodes et al. (2005)] (Rhodes et al., 2005) and modified for studying long‐term ethanol consumption in mice by [Santos et al. (2013)] (Santos et al., 2013). Mice were given access to one bottle of water and one bottle of 20% (v/v) ethanol or 5% (w/v) sucrose in their home cage environment for 2 hr, 3 hr into the dark phase of the light cycle Monday‐Friday for approximately 3 weeks. The lights were turned off at 9 am and drinking sessions commenced at 12 pm. Mice were weighed daily prior to commencement of drinking sessions and bottles were weighed at the start and end of the session to calculate the consumption of each liquid. Preference was calculated as total volume of ethanol or sucrose divided by the total volume of all fluids consumed times 100 for standard 2BC groups, whereas preference for IntelliCage groups were calculated as total number of licks to the ethanol or sucrose supper tubes divided by the total number of licks to all the sipper tubes times 100. For the standard group‐housed mice, the total volume of ethanol or sucrose and water was divided by the number of mice per cage such that the preference for ethanol or sucrose represents the average individual preference per cage. However, the preference for ethanol or sucrose for the individually housed and IntelliCage, is that measured for each mouse. Mice were housed individually (I) or in groups of 3–5 per cage (S) or groups of 12 per cage (IntelliCage, E). After approximately 3 weeks of drinking sessions (18 sessions for ethanol and 15 sessions for sucrose), individually housed mice were group housed (IS), and group‐housed mice were individually housed (SI) (Figure 1). The mice were given 3 days to adapt to the new housing conditions. Drinking sessions continued under these conditions for a further seven sessions. Individually housed mice consuming ethanol were not able to be rehoused in groups in the standard 2BC cages due to excessive aggressive behavior. These mice were instead group housed in the IntelliCage (IE) and vice versa (EI). For sucrose, 18 mice were group housed in six cages and 18 mice were individually housed. For ethanol, 27 mice were group housed in nine cages and 12 mice were individually housed.

### 2.4 IntelliCage 2BC

The IntelliCage is an automated device which can track the individual behaviors of up to 16 mice simultaneously via subcutaneously implanted transponders. The IntelliCage (20 × 55 × 38 cm) contained four standard cubby houses in the center of the arena and four automated cubby houses, one per corner. Antennas at the entrance to each automated corner allowed the IntelliCage to track how many visits each mouse made to each corner and how long they spent in each corner via the transponders. A temperature sensor confirmed that the mouse was present in the corner and infrared beams monitored nose‐pokes to each drinking port. A lickometer recorded the number of licks to each sipper tube. The automated cubby houses also contained access holes for two bottles, with cue lights above the access holes. Each mouse had a transponder implanted subcutaneously, under isofluorane anesthesia, between the shoulder blades 3 days before drinking sessions commenced. During the nontest period, the access port to the ethanol/sucrose bottle was closed and the cue lights were off. The room lights were on a 12 hr reverse light cycle and turned off at 11 am. The drinking sessions commenced at 2 pm, at which point the cue lights turned on and the door to the ethanol/sucrose port opened. Two hours later, the access port to the ethanol/sucrose bottle was closed and the cue lights turned off. Each day, the port for the ethanol/sucrose bottle was alternated with the water port immediately prior to the commencement of the drinking session. The access port to the water bottle remained open and the mice could freely access any of the four automated corners at all times. Preference for ethanol or sucrose was calculated as the percentage of the number of licks to the ethanol or sucrose bottles divided by the total number of licks to the ethanol or sucrose and water bottles. Twelve mice were housed in the IntelliCage for the ethanol experiments and 12 for the sucrose experiments. Due to computer malfunction, the data from days 7, 9, and 13–15 for E mice consuming sucrose (Figure 4) were not saved. The computer malfunction did not affect the operation of the IntelliCage, only the transfer of data, and as such access to sucrose during the drinking session on these days was not affected.

### 2.5 Statistics

Statistical analysis was performed using GraphPad Prism software (version 6, USA) and all results are expressed as mean ± standard error of the mean (SEM). Ethanol and sucrose preference under the different housing conditions were compared using ordinary two‐way ANOVA with Bonferroni's post hoc test, except when analyzing the effect of switching the housing conditions in the isolated to social and social to isolated groups that were drinking ethanol. A one‐way ANOVA with Bonferroni's post hoc test was used as data could not be collected for the post‐housing switch period for the isolated to social group. For the analysis of the data presented in Figure 4, all the data collected on days 7, 9, and 13–15 (corresponding to the IntelliCage computer malfunction for sucrose‐consuming mice) were excluded from the analysis.

## 3 RESULTS

### 3.1 The effect of social and environmental enrichment on ethanol consumption

**Figure 2:**
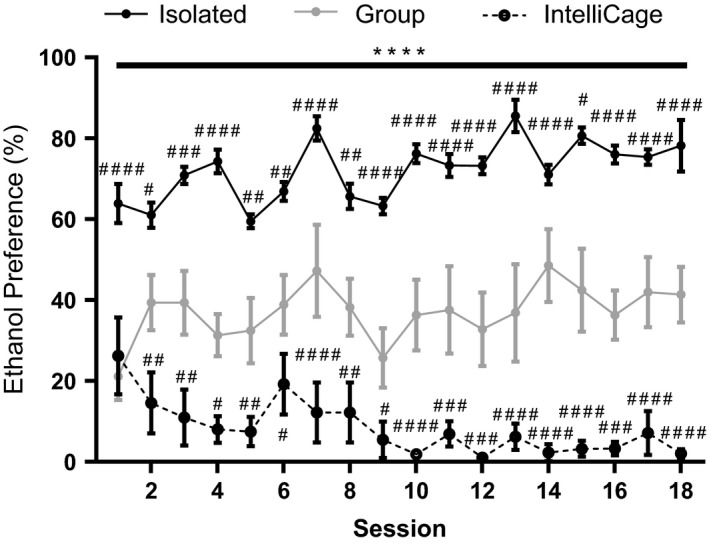
Ethanol preference decreases as the amount of social and environmental enrichment increases. Mice housed individually (I, black dots) had a greater preference for ethanol across all drinking sessions than mice housed in groups (S, gray dots) or the IntelliCage (E, black dots, dashed line). Mice housed in the IntelliCage had the lowest preference for ethanol. One‐way ANOVA with Bonferroni's post hoc test. ****p < .0001 compared to IntelliCage. ^#^ *p* < .05, ^##^ *p* < .01, ^###^ *p* < .001, ^###*#*^ *p* < .0001 compared to social

To determine whether ethanol consumption could be modulated by manipulating social and environmental conditions, we compared the preference for ethanol over water from mice living in single housing without enrichment (I), group housing without enrichment (S), and group housing with enrichment (IntelliCage, E). As the types of bottles used in the IntelliCage differed from the bottles used in the standard cages (300 ml capacity, vertical placement without ball bearings vs. 50 ml capacity, angled placement with double ball bearings), we used preference for ethanol over water rather than ethanol consumption (g/kg) to compare consumption behaviors under the different housing conditions. For the I and S cages, preference was amount of ethanol consumed (ml) as a percentage of the total amount of all fluids (ml) consumed per mouse and for E, the preference was the number of licks to the ethanol sipper tubes as a percentage of the total number of licks to all sipper tubes. Two‐way ANOVA revealed a significant interaction of drinking session and housing condition on ethanol consumption (*F*(34,536) = 1.792, *p* = .0045) and an effect of housing condition (*F*(2,536) = 673.6, *p* < .0001) but no effect of drinking session alone (*F*(17,536) = 1.3, *p* = .1863). Bonferroni's post hoc analysis showed I mice housed had higher preference for ethanol than both the S and E mice on all drinking days (*p* < .05–0.0001) (Figure 2). On all but the first day of ethanol consumption, S mice had a higher preference for ethanol than the E mice (*p* < .05–.0001). The average preference for ethanol over the 18 drinking sessions was 65.20 ± 1.747%, 37.09 ± 1.594%, and 8.35 ± 1.570% for I, S, and E mice, respectively.

### 3.2 Switching from a deprived to enriched environment reduces ethanol preference

**Figure 3:**
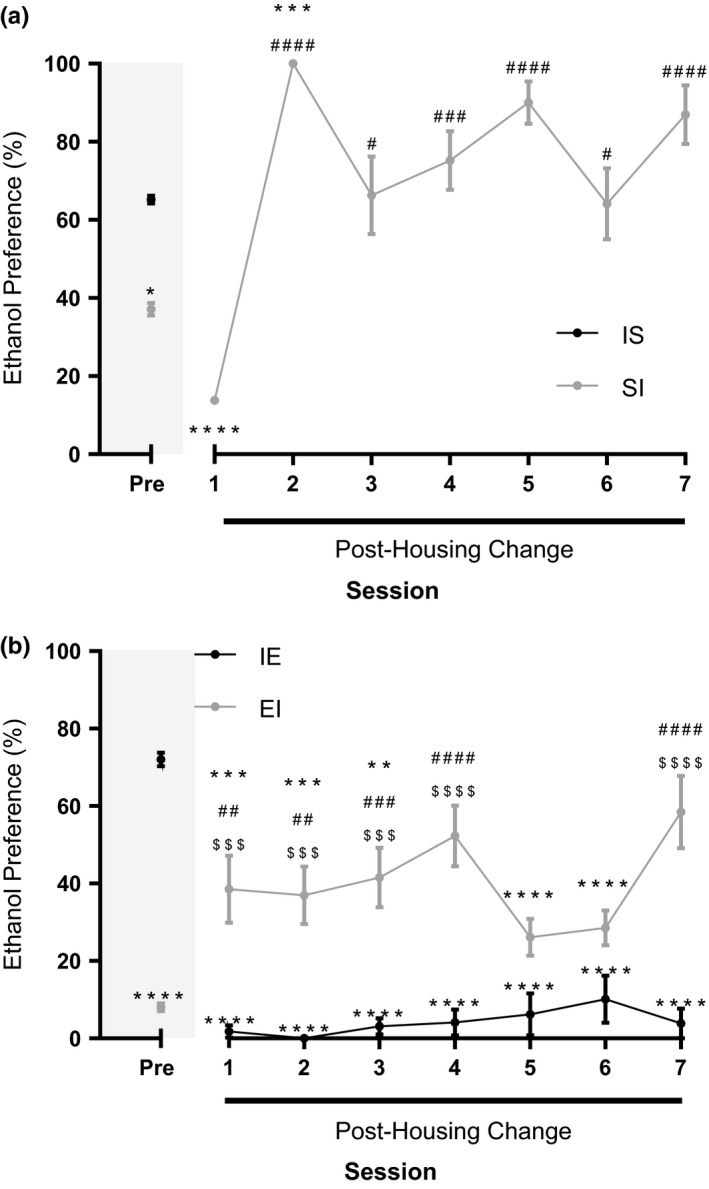
Moving from an enriched to a deprived environment increases ethanol preference. (a) Mice moved from social to isolated housing (SI, gray dots) increased their preference for ethanol. The increase in preference was similar to isolated mice (I, black dots) before the housing conditions were changed (Pre, sessions 1–18). (b) There was an increase in preference when mice were moved from the IntelliCage to isolated housing (EI, gray dots) and a decrease in preference when moved from the isolation to the IntelliCage (IE, black dots). Pre‐housing condition change (sessions 1–18), EI mice had a lower preference for ethanol compared to IE mice. Post‐housing condition change their ethanol preference increased and was similar to the pre‐housing change preference of IE mice on days 22 and 25. When mice were moved from I to E, they reduced their ethanol preference, similar to the pre‐housing change ethanol preference (sessions 1–18) of EI mice. Two‐way ANOVA with Bonferroni's post hoc test. *p < .05, **p < .01, ***p < .001, ****p < .0001 compared to IE Pre. ^#^p < 0.05 compared to EI pre, ^##^ *p* < .01, ^###^ *p* < .001, ^####^ *p* < .0001 compared to EI Pre. ^$$$^ *p* < .001, ^$$$$^ *p* < .0001 compared to IE sessions 19–25

As the mice in the previous experiment were naïve to ethanol and prior ethanol experience can alter the perceived reward value of ethanol (Shimizu, Oki, Mitani, Nakamura, & Nabeshima, 2015), we explored whether the biological effects of ethanol outweighed the effects of social and environmental enrichment in mice with previous ethanol experience. To achieve this, we provided enrichment to deprived mice and deprived enriched mice of social and environmental interactions. We then compared their average ethanol preference in the new and old housing conditions. We rehoused the I mice into groups of three per cage (IS) and the S mice into individual cages (SI) and allowed the mice 3 days to habituate to the new conditions before drinking session recommenced. However, we were unable to recommence the drinking session for the isolated to social housing mice due to aggressive behavior. Therefore, we compared the average ethanol preference of the IS mice prior to changing housing conditions with the ethanol preference of the SI mice before and after the housing changes (Figure 3a). Using one‐way ANOVA with Bonferroni's post hoc test, we found that removal of social interaction caused a significant increase in ethanol preference (*F*(8,132)=21.49, *p* < .0001) from the second drinking session after changing the housing conditions. The average ethanol preference of the SI mice prior to changing the housing conditions was lower than the IS mice (*p* < .05). Following the change in housing conditions, the ethanol preference of the SI mice differed from the average ethanol preference of the IS mice only on the drinking session immediately following the housing change (*p* < .001). The average ethanol preference in the isolated to social group prior to the housing change was 65.20 ± 1.747% and for the social to isolation groups the average ethanol preference was 37.09 ± 1.594% and 70.92 ± 10.710% before and after the housing switch, respectively.

To reduce aggressive behavior and enable examination of ethanol preference in a social setting, we moved a second group of I mice (average pre‐housing switch preference: 72.07 ± 1.761%) to the IntelliCage and E mice into individual housing (Figure 3b). Comparison of the average pre‐ and post‐housing change in ethanol preference between both groups of mice was measured using two‐way ANVOA with Bonferroni's post hoc test. As shown in the experiment above, prior to the change in housing conditions, the average ethanol preference of the IE mice was higher than that of the EI mice (*p* < .0001). There was a significant interaction of drinking session and housing condition on ethanol preference (*F*(7,186)=25.27, *p* < .0001) with an effect of drinking session (*F*(7,186)=4.67, *p* < .0001) and housing condition (*F*(1,186)=80.08, *p* < .0001) alone. Prior to the housing change, the average ethanol preference of EI mice was less than IE mice (*p* < .0001). When I mice were rehoused in the IntelliCage they reduced their ethanol preference similar to the ethanol preference of the E mice before their housing conditions were altered. The reduction in preference in the IE mice was significant from the first drinking session after the housing switch (*p* < .0001). Conversely, the EI mice increased their ethanol preference on all but the fifth and sixth session following the housing alteration (*p* < .01 to 0.0001). On the fourth and seventh session, the ethanol preference of the EI mice was similar to the average ethanol preference before the housing change of the IE (*p* > .05). The average ethanol preference of the IE mice was 72.07 ± 1.761 and 4.20 ± 1.228% and the EI mice was 8.35 ± 1.570 and 40.34 ± 4.449% before and after the housing change, respectively.

### 3.3 Social and environmental enrichment increases sucrose consumption in naïve mice

**Figure 4:**
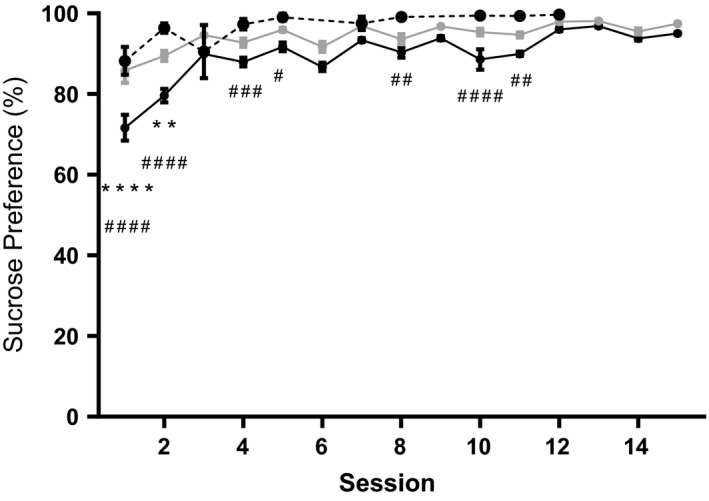
Social and environmental enrichment increases sucrose preference. Mice housed in IntelliCage (E, black dots, dashed line) had a greater preference for sucrose than mice housed in isolation (I, black dots). The sucrose preference of E mice was not different to socially housed mice (S, gray dots). Two‐way ANOVA with Bonferroni's post hoc test. **p < .01, ****p < .0001 compared to S. ^#^ *p* < .05, ^##^ *p* < .01, ^###^ *p* < .001, ^####^ *p* < .0001 compared to E

Previous studies indicate that, unlike ethanol consumption, social and environmental factors may not be as important for sucrose consumption. To investigate this possibility, we replicated the ethanol experiments (above) in a new cohort of mice consuming sucrose. Analysis using two‐way ANOVA found a significant interaction of drinking session and housing condition (*F*(18,324)=2.304, *p* = .0021), an effect of drinking session (*F*(9,324)=12.72, *p* < .0001), and an effect of housing condition alone (*F*(2,324)=59.57, *p* < .0001) on sucrose preference. Bonferroni's post hoc test revealed a significant increase in sucrose preference in S mice (sessions 1 and 2, *p* < .0001 and 0.01, respectively) and E mice (sessions 1–2, 4–5, 8, 10–11: *p* < .05 to *p* < .0001) compared to I mice (Figure 4). There was no difference in sucrose preference between the S and E mice during any of the drinking sessions. The average preference for sucrose over the 15 drinking sessions was 89.71 ± 1.711%, 94.46 ± 0.877%, and 96.68 ± 1.275% for I, S, and E mice, respectively.

### 3.4 Environmental enrichment does not influence preference in sucrose‐experienced mice

**Figure 5:**
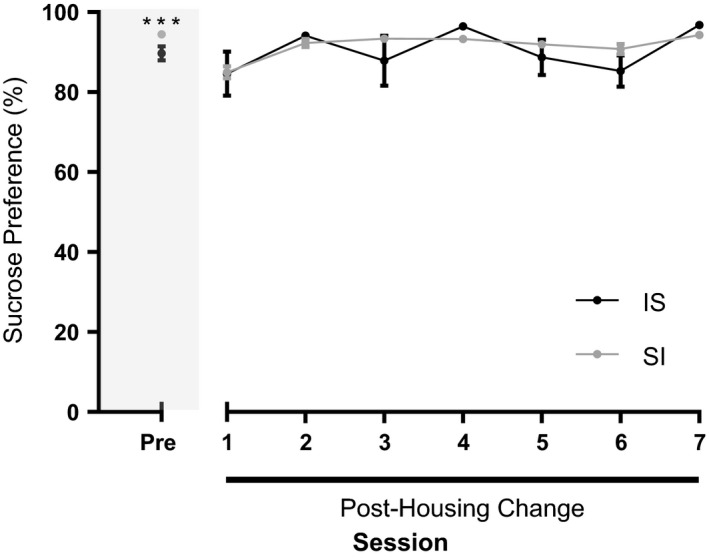
Sucrose preference is not affected when social enrichment is removed. Before housing conditions were changed (Pre, sessions 1–15), isolated to social (IS, black dots) mice had a lower preference for sucrose than social to isolated (SI, gray dots) mice. After the housing change (sessions 16–22), there was no difference in sucrose preferences between the SI and IS groups nor did it differ from the pre‐housing change (sessions 1–15) sucrose preference of either group. Two‐way ANOVA with Bonferroni's post hoc test. ***p < .001 compared to IS mice

Finally, to determine whether the removal or addition of social interaction impacts sucrose preference in mice with previous sucrose experience, we rehoused I mice in groups of three per cage (IS) and S in individual cages (SI) (Figure 5). As the aggressive behavior demonstrated by the ethanol mice was not observed in the sucrose mice, and we found no difference in sucrose preference in S and E mice in the previous experiment, we recommenced the drinking sessions in both groups of mice after a 3‐day habituation period using standard cages only. Analysis with two‐way ANOVA and Bonferroni's post hoc test showed no interaction of drinking session and housing condition on sucrose preference (*F*(7,172)=2.005, *p* = .057), no effect of housing condition (*F*(1,172)=2.481, *p* = .117) but an effect of drinking session alone (*F*(7,172)=7.357, *p* < .0001). The average sucrose preference of the IS mice was 89.71 ± 1.711 and 90.57 ± 1.956% and the SI mice was 94.46 ± 0.877 and 91.56 ± 1.183% before and after the housing switch, respectively.

## 4 DISCUSSION

In this study, we examined the effect of social and environmental enrichment on ethanol and sucrose consumption. We show that ethanol preference increases with social and environmental deprivation and that this increase in preference can be reversed with the provision of social and environmental enrichment. Additionally, we found that ethanol preference is inversely proportional to the level of environmental enrichment provided implying social and cognitive interactions are more rewarding than the pharmacological effects of ethanol. Our findings for ethanol preference are consistent with previous publications (see Table 1) and a study by Sampson and colleagues showing that rats are willing to do more work to obtain a sucrose reward compared to an ethanol reward (Samson, Slawecki, Sharpe, & Chappell, 1998). Pang and colleagues demonstrated that the provision of social or environmental enrichment reduces ethanol consumption in female C57BL/6 mice (Pang et al., 2013). Wolffgramm and colleagues produced a similar result in male Wistar rats (Wolffgramm, 1990). They allowed sensory contact (sight, smell, vocal communication) without physical contact by placing a perforated divider in the cage and housing a rat on either side of the divider. The rats with sensory contact consumed more ethanol than group‐housed rats but less than individually housed rats.

Conversely, Rockman and colleagues found the concurrent addition of social and environmental enrichment increased ethanol consumption in Wistar and Sprague Dawley rats (Rockman et al., 1989; Rockman, Borowski, & Glavin, 1986). Extending on these findings, they showed that rats moved to an enriched environment drank similar amounts of ethanol to rats remaining in isolation and more than the rats moved from an enriched to a deprived environment, while the rats remaining in the enriched environment drank the most ethanol (Rockman et al., 1989). Interestingly, a study by Juarez and Vazquez‐Cortes showed that when ethanol is offered in an isolated setting, rats normally housed I, S, IS, or SI drink similar amounts of ethanol (Juarez & Vazquez‐Cortes, 2003). When ethanol was consumed in a social setting, all the rats, except SI, drank similar amounts of ethanol. The SI group drank more ethanol than any of the other groups.

While the later studies appear to conflict with our findings and the previous studies (demonstrating rodents in isolation consume more ethanol than socially housed animals), further examination of the methodology used indicates that they may actually support the finding of this and former studies. The studies by Rockman and Juarez and Vazquez‐Cortes, all involved altering the housing environments of ethanol‐experienced rats. In each of these studies, rehousing ethanol‐experienced rats in a social setting produced an increase in ethanol consumption; whereas the former studies were performed in ethanol‐naive animals. From previous rodent research, we know that changes to housing conditions are stressful for rodents (Misslin, Herzog, Koch, & Ropartz, 1982; Tuli, Smith, & Morton, 1995), rodents in an isolated housing environment are more sensitive to stress than those in a social environment (Giralt & Armario, 1989), stress alters ethanol consumption (Cozzoli, Tanchuck‐Nipper, Kaufman, Horowitz, & Finn, 2014; Meyer, Long, Fanselow, & Spigelman, 2013), ethanol experience alters ethanol reward value (Shimizu et al., 2015; McCusker & Bell, 1988), and ethanol consumption reduces the ability to cope with stress (Zhao, Weiss, & Zorrilla, 2007). Taken together, one could argue that for ethanol‐naïve rats, the provision of social interactions provides greater rewarding benefits/stress relief than the biological effects obtained from ethanol consumption. But for ethanol‐experienced rats, moving into a socially and cognitively enriched setting appears to be stressful, and the rewarding benefits/stress relief from the biological effects of ethanol is perceived to outweigh those obtained from engaging in social and environmental interactions.

It is difficult to say whether the same could be said for mice. Certainly, we were unable to rehouse ethanol‐experienced mice into groups due to aggressive behavior, and it could be that the aggressive behavior indicated that the ethanol‐experienced mice had a reduced capacity to cope with the changes in housing conditions since stress can increase aggressive behavior in mice (Yang et al., 2015). However, we were able to rehouse the ethanol‐experienced mice in a social environment with the IntelliCage. Most likely, the additional space per animal allowed greater capacity to escape aggressive/stressful challenges and the cognitive stimulation provided distraction from the stress created by the change to housing conditions. However, further studies are required to explore these possibilities.

In this study, we also demonstrate that social and environmental enrichment plays a different role in sucrose and ethanol consumption. Increasing enrichment produced a reduction in ethanol preference in both the naïve and ethanol‐experienced animals. However, in naïve mice, providing enrichment increased sucrose consumption, whereas the amount of enrichment provided had no effect on sucrose preference in sucrose‐experienced mice. Broadly, consumption behaviors can be broken down into different phases: acquisition, maintenance, withdrawal, and relapse (for reviews see, Bhutada et al., 2012; Carroll, 1993; Marchant, Li, & Shaham, 2013; Robinson, Khurana, Kuperman, & Atkinson, 2012). In this study, the ethanol preference of the naïve mice was measured during the acquisition and maintenance phases, whereas the ethanol preference of the experienced mice was measured during the maintenance phase only. This suggests that social and environmental enrichment may be important for acquisition but not maintenance of sucrose consumption; differing from ethanol consumption where both acquisition and maintenance were modulated by social and environmental enrichment. It also suggests that (in these circumstances) the reward benefits/stress relief gained from sucrose consumption outweigh those obtained from social and environmental interaction, particularly during the maintenance phase of sucrose consumption. That is, sucrose may be more addictive than ethanol. In support of this hypothesis, Sampson and colleagues measured the breakpoint in rats responding for ethanol and sucrose (Samson et al., 1998). They found that a higher percentage of rats would press the lever at breakpoint (32 lever presses for a single reward delivery) for sucrose (~80%) than ethanol (~40%).

Along the same lines, it is also possible that greater levels of stress are required to increase sucrose consumption during the maintenance phase. Supporting this, Grimm and colleagues (Grimm et al., 2013) found that following 29 days of withdrawal from sucrose, rats which experienced social interactions lever pressed for sucrose more than rats housed in isolation. Additionally, Gill and Cain demonstrated that under normal feeding conditions, environmentally enriched Sprague Dawley rats lever pressed for sucrose rewards less than those housed individually (Gill & Cain, 2011). However, under more stressful conditions (when the rats were deprived of food) the environmentally enriched rats pressed for sucrose more than the deprived rats.

Interestingly, McCool and Chappell found socially housed Long Evans rats’ lever pressed for sucrose more than isolated rats, but following a single day of extinction training, no difference could be found (McCool & Chappell, 2009). The difference between the studies by Gill and Cain and McCool and Chappell may lie in the delivery methods chosen. While both studies used operant procedures to deliver sucrose, McCool and Chappell provided access to a sipper tube for 20 s for each successful response for sucrose compared to the more traditional delivery method used by Gill and Cain (dipper cup with 0.1 ml sucrose for 4 s). Given the similarity of McCool and Chappell's finding to those presented in this study, the provision of a sipper tube may have created a drinking environment more akin to the IntelliCage than that of a traditional operant self‐administration setting. Together, these studies highlight the importance of considering the methodology used and motivational state of the animal when interpreting the findings of consumption studies.

In another study, Van den Berg and colleagues demonstrated that single‐housed rats have greater conditioned place preference for sucrose than socially housed rats (Van den Berg et al., 1999). These rats did not have prior experience with sucrose, nor were they provided with enrichment or subjected to stress, which could explain why these findings are similar to sucrose responding under non‐food deprived conditions (Gill & Cain, 2011). Experiments exploring the effects of stress on conditioned place preference in sucrose‐naïve and ‐experienced rat housed in enriched and deprived environments are required to clarify how these factors influence sucrose consumption behaviors. However, it is clear that the relationship between social and environmental interactions and consumption behaviors is complex and more research is required to understand how these factors are specifically involved in sucrose consumption.

It is noteworthy that in this study, the mice (regardless of the housing conditions) consume sucrose almost exclusively when offered a choice between sucrose and water. Srisontiyakul and colleagues have shown that in order to match lever responding in an operant setting for 10% ethanol, they had to reduce the concentration of sucrose from 5% to 0.3–1% (Srisontiyakul, Kastman, Krstew, Govitrapong, & Lawrence, 2016). The literature on sucrose consumption is quite diverse, with reported sucrose concentrations used for consumption studies varying from 0.3% to 35%. The most commonly reported concentrations are 5 and 10% sucrose. We chose 5% over 10% based on Srisontiyakul's study, to allow comparisons with our previous publications showing that long‐term consumption of 5% sucrose using the IA2BC model alters nucleus accumbens morphology (Klenowski, Shariff, et al., 2016) and our studies showing the 5% concentration can be used to test novel compounds for their ability to reduce binge‐like sucrose consumption (Steensland et al., 2010; Patkar et al., 2017; Nielsen et al., 2008; Shariff et al., 2016; Simms, Haass‐Koffler, Bito‐Onon, Li, & Bartlett, 2012; Simms et al., 2011; Srinivasan et al., 2012). While it is possible that the preference for sucrose represents a celling value and reducing the sucrose concentration may enable further dissection of the factors which lead to increased consumption of sucrose, it is more likely that sucrose consumption, regardless of the concentration consumed, is not significantly modulated by social and environmental conditions. Hall and colleagues found socially and individually housed rats consumed similar amounts of 0.7, 2.1, 7.0, 21.0, and 34.0% sucrose solutions (Hall et al., 1997). However, this aspect remains to be explored.

It is also important to address the reduced ethanol consumption of EI mice during sessions 5 and 6 post housing switch (Figure 3a). It is unclear whether this reduction is an artifact or a reflection of biological or social structure changes. Based on previous studies within our laboratory, it is not unusual during the first 2 weeks of ethanol exposure to see ethanol‐inexperienced mice, which initially consume ethanol at high levels, reduce their consumption for a day or two before returning to high levels of consumption. A similar dip in ethanol consumption can also be seen in Figure 1 of Rhodes’ study (Rhodes et al., 2005) and in figure 2A of Matsons’ study (Matson, Kasten, Boehm, & Grahame, 2014). Together, this implies that the reduced consumption is most likely biological in nature and unrelated to disturbances within the animal facility or experimental protocol. However, it is unclear what mechanisms may underlie this phenomenon. The most likely explanation is that the mice (temporarily) reduce their ethanol consumption due to the negative/aversive effects produced by repetitive daily binge‐like ethanol consumption prior to the development of tolerance. Supporting this possibility, Carnicella and colleague demonstrated a u‐shaped ethanol dose response curve in rats’ lever pressing for access to ethanol (Carnicella, Yowell, & Ron, 2011). Specifically, the rats progressively increased their response for ethanol until the ethanol concentration reached 10–20%, and as the concentration increased beyond this point, their responding progressively decreased. Additionally, a study by Linsenbart and colleagues shows that mice consuming ethanol using the DID model had more hind foot slips on the balance beam on day 8 than mice consuming water; but by day 15, the number of hind foot slips was similar to water‐consuming mice (Linsenbardt, Moore, Griffin, Gigante, & Boehm, 2011), suggesting that the mice developed tolerance to ethanol sometime before the 15th day of exposure.

It should also be noted that we included a habituation period prior to commencing DID sessions and immediately following changes to housing conditions. The mice did not have access to their reinforcer during this time and it is possible that including a habituation period may have masked an effect of the housing switch, especially for sucrose intake. Certainly, there would be effects of the condition change on consumption during this period, had the mice has access to their reinforcer. However, it would be extremely difficult to dissect these effects from those produced by novelty and behaviors related to the reestablishment of social hierarchies. In this study, we chose to include a habituation period for three reasons. The first is primarily an ethical consideration: we wanted to ensure the wellbeing of the animals. Taking rodents from social to isolated housing and from isolation into a social environment is very stressful and we needed to ensure there were no adverse wellbeing effects which could reduce consumption and confound our interpretation of the results. Secondly, we did not want the behaviors related to establishment of new social hierarchy, responses to novelty, the potential for fretting and appetite disturbances, and so on, produced by the initial change in housing conditions to interfere with the reestablishment of consumption behaviors. Thirdly, we gave the mice an adaption period prior to commencing the pre‐housing change drinking sessions and, as a control, wanted to match the conditions of the pre‐ and post‐housing change drinking period as closely as possible. While the experimental conditions of this study were not suitable for studying consumption behaviors immediately after the housing switch, it would be possible to examine these aspects by making more subtle changes to the housing environment, like gradually increasing or decreasing the size of the cage, the number of mice in the cage or the amount of enrichment provided. It will be interesting to see the results of such studies in the future.

Research has shown that long‐term binge‐like consumption of ethanol (Klenowski, Fogarty, et al., 2016) and sucrose (Klenowski, Shariff, et al., 2016) alter the structure of the brain and exposure to adverse environments during childhood significantly increase the risk of developing alcoholism and obesity (and other disorders) in later life (Brady & Back, 2012; Daoura, Haaker, & Nylander, 2011; Enoch, 2012). Certainly, epidemiological studies show that those living in a lower socioeconomic environments are more likely to be impacted by harmful alcohol consumption and/or obesity (Batty, Lewars, Emslie, Benzeval, & Hunt, 2008; McCartney et al., 2016; Ferraro, Schafer, & Wilkinson, 2016; Non et al., 2016). While the mechanisms underlying the detrimental neural effects of ethanol and sucrose remain unclear, the provision of environmental enrichment has been shown to be neuroprotective in rodents (Anastasía, Torre, de Erausquin, & Mascó, 2009; Barone, Novelli, Piano, Gargini, & Strettoi, 2012; Griñan‐Ferré et al., 2016; Kiss et al., 2013; Livingston‐Thomas et al., 2016). Although the results from human studies neither support nor refute the neuroprotective benefits of environmental enrichment, research has recently become focused on developing cognitive training, such as working memory and mindfulness exercises, for attenuating and/or preventing neuroplastic changes associated with these disorders (Rogers, Ferrari, Mosely, Lang, & Brennan, 2017; Ruffault et al., 2016; Chiesa & Serretti, 2014; Karyadi, VanderVeen, & Cyders, 2014; Lee, An, Levin, & Twohig, 2015; Tang, Tang, & Posner, 2016; Bega, Gonzalez‐Latapi, Zadikoff, & Simuni, 2014; Corbett, Jeffers, Nguemeni, Gomez‐Smith, & Livingston‐Thomas, 2015; Milgram, Siwak‐Tapp, Araujo, & Head, 2006; Zigmond & Smeyne, 2014).

## 5 CONCLUSIONS

The IntelliCage system was a useful and versatile device for examining the involvement of social and environmental enrichment in consumption behaviors in mice. We demonstrated that the addition of social and environmental enrichment had different effects on ethanol and sucrose consumption behaviors in male C57BL/6 mice. The addition of enrichment reduced ethanol consumption in ethanol‐naïve and ‐experienced mice and increased sucrose consumption in sucrose‐naïve but not ‐experienced mice. While we can take advantage of these differences to advance our understanding of the mechanisms involved in the different phases of consumption disorders, we also need to carefully consider these factors when developing novel therapies. The behavioral characteristics of sucrose consumption are different from ethanol consumption, suggesting that treatment approaches similar to that used for AUDs may not be appropriate for controlling excessive sugar consumption.

## CONFLICT OF INTERESTS

None declared.
